# Identification of Key Performance Indicators for Hospital Management Using an Extended Hesitant Linguistic DEMATEL Approach

**DOI:** 10.3390/healthcare8010007

**Published:** 2019-12-25

**Authors:** Ling Zhang, Ran Liu, Shan Jiang, Gang Luo, Hu-Chen Liu

**Affiliations:** 1 Faculty of Engineering and Information Technology, University of Technology Sydney, Broadway, NSW 2007, Australia; lingzhangshu@foxmail.com; 2 SILC Business School, Shanghai University, Shanghai 200444, China; gluo@shu.edu.cn; 3 School of Management, Shanghai University, Shanghai 200444, China; ranliu@shu.edu.cn; 4 College of Economics and Management, China Jiliang University, Hangzhou 310018, China; huchenliu@foxmail.com

**Keywords:** healthcare management, key performance indicator, double hierarchy hesitant fuzzy linguistic term set, DEMATEL technique, TOPSIS method

## Abstract

Performance analysis is of great significance to increase the operational efficiency of healthcare organizations. Healthcare performance is influenced by numerous indicators, but it is unrealistic for administrators to improve all of them due to the restriction of resources. To solve this problem, we integrated double hierarchy hesitant fuzzy linguistic term sets (DHHFLTSs) with the decision-making trial and evaluation laboratory (DEMATEL) and proposed a DHHFL– DEMATEL method to identify key performance indicators (KPIs) in healthcare management. For the developed approach, the judgments of experts on the inter-relationships among indicators were represented by DHHFLTSs, and a novel combination weighting approach was proposed to obtain experts’ weights in line with hesitant degree and consensus degree. Then, the normal DEMATEL method was extended and used for examining the cause and effect relationships between indicators; the technique for the order of preference by similarity to the ideal solution (TOPSIS) method was utilized to generate the ranking of performance indicators. Finally, the feasibility and effectiveness of the proposed DHHFL–DEMATEL approach were illustrated by a practical example in a rehabilitation hospital.

## 1. Introduction

Over the past decades, there has been great change in the construction of healthcare systems [1,2]. Due to the growing demands for quality improvement and the increasing pressure from regulatory agencies, problems on how to provide high-quality medical services and improve patient satisfaction have caught the attention of hospital administrators [3,4]. The assessment and measurement of healthcare performance play a great part in hospital management and can significantly affect the operational efficiency of a healthcare management system [5,6]. It is essential to determine the performance indicators that influence the overall system and have a great potential in obtaining high-quality performance [7,8,9]. However, due to the constraint of hospital resources, it is unrealistic to optimize all performance indicators at the same time. In this regard, it is favorable to focus on urgent and important indicators, i.e., key performance indicators (KPIs), and to improve them step by step [10]. 

In prior researches, KPIs of healthcare management were generally determined via prepared questionnaires and expert interviews [1,10]. Nevertheless, few studies considering KPIs in healthcare management divided them into meaningful indicators and investigated the relationships among them [2,7]. Thus, it is necessary to analyze the inter-relationships between performance indicators and determine KPIs to measure and monitor the performance of a healthcare organization. The decision-making trial and evaluation laboratory (DEMATEL) is an effective technique developed by Gabus and Fontela [11] to analyze the causal relationships between elements [12,13,14]. This method can visualize the structure of complicated causal relationships through matrixes or digraphs [15,16]. Because of its advantages and capabilities, the DEMATEL has been widely applied in various fields, such as emergency management [9,17,18], supply chain management [19,20,21], waste management [22,23], quality function deployment [24], failure mode risk analysis [15,25], re-distributed manufacturing [26], and sustainability performance assessment [27,28].

In practical applications, it is usually difficult for experts to estimate the direct effects between elements with crisp numbers or simple linguistic terms because of the imprecise and vague nature of human judgments [29,30]. To describe both fuzziness and randomness of uncertain linguistic information provided by experts, Gou et al. [31] proposed the concept of double hierarchy hesitant fuzzy linguistic term set (DHHFLTS). The DHHFLTS consists of two hierarchy linguistic terms: the first one is used for describing the general evaluation information of experts, while the second one is a supplemental linguistic term set to modify the term of the first one [31,32]. This method can be deemed as an effective method to describe the linguistic evaluation information of decision makers comprehensively [33]. Up to now, the DHHFLTSs have been adopted by many researchers to handle complex linguistic evaluation information. For example, Duan et al. [34] used the DHHFLTSs and *k*-means clustering to assess and cluster the risk of failure modes. Liu et al. [35] proposed a double hierarchy hesitant fuzzy linguistic PROMETHEE (preference ranking organization method for enrichment evaluation) method to evaluate public-private-partnership’s advancement. Wang et al. [36] presented a double hierarchy hesitant fuzzy linguistic ORESTE (organísation, rangement et Synthèse de données relarionnelles, in French) approach for the assessment of traffic congestion. Gou et al. [31] evaluate the implementation status of haze controlling measures with MULTIMOORA (multi-objective optimization by ratio analysis plus the full multiplicative form) method under double hierarchy hesitant fuzzy linguistic environment. Montserrat-Adell et al. [37] presented an extended version of DHHFLTSs, called free double hierarchy hesitant fuzzy linguistic term sets, and applied it to a group decision making problem involving tourist attractions. 

In this paper, we develop an integrated model combining DHHFLTSs with the DEMATEL method to analyze the interrelationships among healthcare performance indicators and identify KPIs for hospital management. Specifically, we utilize the DHHFLTSs to represent the opinions of experts on the influential interrelationships among performance indicators, to address their uncertainty and vagueness. The DEMATEL method is modified and employed to analyze the complex interrelationships of indicators and obtain healthcare KPIs. In addition, a modified technique for the order of preference by similarity to the ideal solution (TOPSIS) method is proposed for ranking and screening the given performance indicators. Finally, an empirical illustrative study is presented to prove the effectiveness and efficiency of the proposed DHHFL–DEMATEL approach. 

The remaining sections of this article are arranged as follows. In Section 2, some basic definitions and operations of DHHFLTSs are introduced. In Section 3, we develop the DHHFL– DEMATEL model to identify KPIs in hospital management. Section 4 provides a case of a rehabilitation hospital in China to demonstrate the proposed combined approach. Finally, we conclude this study and provide further research recommendations in Section 5.

## 2. Preliminaries

### 2.1. Double Hierarchy Linguistic Term Sets

The double hierarchy linguistic term sets (DHLTSs) were developed by Gou et al [31] for expressing complex linguistic information.

**Definition** **1** **([31]).**  *Suppose that*$S=\left\{s_{t}|t=-\tau ,\ldots ,-1,0,1,\ldots ,\tau \right\}$*and*$O=\left\{o_{k}|k=-\varsigma ,\ldots ,-1,0,1,\ldots ,\varsigma \right\}$*are the first and second hierarchy linguistic term sets, respectively, and they are completely independent. Then a DHLTS S_O_ can be defined as follows:*

(1) $$S_{O}=\left\{s_{t<O_{k}>}|t=-\tau ,\ldots ,-1,0,1,\ldots ,\tau ;k=-\varsigma ,\ldots ,-1,0,1,\ldots ,\varsigma \right\}$$

*where*$s_{t<O_{k}>}$*is called a double hierarchy linguistic term, and*$o_{k}$*represents the second hierarchy linguistic term when the first hierarchy linguistic term is*$s_{t}$.

If t≥0, it indicates that the first hierarchy linguistic term set $S=\left\{s_{t}\left|t\geq 0\right.\right\}$ is non-negative; so the second hierarchy linguistic term set should be described in an ascending order. On the contrary, if t<0, the second hierarchy linguistic term set should be described in a descending order. Moreover, if t=τ, then we only use the front half of the second hierarchy linguistic term set, i.e., $O=\left\{o_{k}\left|k=-\varsigma ,\ldots ,-1,0\right.\right\}$ to describe $s_{\tau }$; and if t=−τ, $O=\left\{o_{k}\left|k=0,1,\ldots ,\varsigma \right.\right\}$ is employed to describe $s_{-\tau }$.

### 2.2. Double Hierarchy Hesitant Fuzzy Linguistic Term Sets

To more accurately and reasonably express experts’ linguistic expressions, Gou et al. [31] extended the DHLTSs into hesitant fuzzy environment and proposed the DHHFLTSs.

**Definition** **2** **([38]).**  *Let X be a fixed set, and*$S_{O}=\left\{s_{t<O_{k}>}|t=-\tau ,\ldots ,-1,0,1,\ldots ,\tau ;\right.$$\left.k=-\varsigma ,\ldots ,-1,0,1,\ldots ,\varsigma \right\}$*be a DHLTS. A DHHFLTS on X,*$H_{S_{O}}$*, is in terms of a membership function that when applied to X returns a subset of*$S_{O}$, *which can be described by*

(2) $$H_{S_{O}}=\left\{\langle x_{i},h_{S_{O}}\left(x_{i}\right)\rangle \left|x_{i}\in X\right.\right\},$$

*where*$h_{S_{O}}\left(x_{i}\right)$*is a collection of values in*$S_{O}$*, denoting the possible membership degrees of the element*$x_{i}\in X$*to the set*$H_{S_{O}}$*. That is,*

(3) $$h_{S_{O}}\left(x_{i}\right)=\{\begin{array}{l} s_{\phi_{l}\langle o_{\varphi_{l}}\rangle }(x_{i})\left|s_{\phi_{l}\langle o_{\varphi l}\rangle }\in S_{O};\;l=1,2,\ldots ,L;\;\phi_{l}=-\tau ,\ldots ,-1,0,1,\ldots ,\tau ;\right.\\ \varphi_{l}=-\varsigma ,\ldots ,-1,0,1,\ldots ,\varsigma \}, \end{array}$$

*with L being the number of the DHLTS in*$h_{S_{O}}\left(x_{i}\right)$*, and*$s_{\phi_{l}\langle o_{\varphi l}\rangle }\left(x_{i}\right)\left(l=1,2,\ldots ,L\right)$*of each*$h_{S_{O}}\left(x_{i}\right)$*is the continuous terms of*$S_{O}$.

For the sake of convenience, we call $h_{S_{O}}\left(x_{i}\right)$ a double hierarchy hesitant fuzzy linguistic element (DHHFLE), and the DHLTS included in a DHHFLE is ranked in ascending order.

**Definition** **3** **([31]).**  *Let* $S_{O}$ *be a DHLTS,* $h_{S_{O}}\left(x_{i}\right)$ *be a DHHFLE containing L linguistic terms in* $h_{S_{O}}$ *, and* $h_{\gamma }=\left\{\gamma_{l}\left|\gamma_{l}\in \left[0,1\right]\;;l=1,2,\ldots ,L\right.\right\}$ *be a hesitant fuzzy element. Then, the membership degree* $\gamma_{l}$ *and* *the subscript* $\left(\phi_{l},\varphi_{l}\right)$ *of the DHLT* $s_{\phi_{l}\langle o_{\varphi_{l}}\rangle }$ *can be converted into each other by the following functions:*

(4) $$\begin{array}{l} f:\left[-\tau ,\tau \right]\times \left[-\varsigma ,\varsigma \right]\to \;\left[0,1\right]\\ f\left(\phi_{l},\varphi_{l}\right)=\left\{\begin{array}{l} \frac{\varphi_{l}+\left(t+\phi_{l}\right)\varsigma }{2\varsigma \tau }=\gamma_{l},\;\;\;if\;-\tau +1\leq \phi_{l}\leq \tau -1,\\ \frac{\varphi_{l}+\left(t+\phi_{l}\right)\varsigma }{2\varsigma \tau }=\gamma_{l},\;\;\;if\;\phi_{l}=\tau ,\;\\ \frac{\varphi_{l}}{2\varsigma \tau }=\gamma_{l},\;\;\;if\;\phi_{l}=-\tau ,\; \end{array}\right. \end{array}$$

(5) $$\begin{array}{l} f^{-1}:\;\left[0,1\right]\to \left[-\tau ,\tau \right]\times \left[-\varsigma ,\varsigma \right],\\ f{-}^{1}(\gamma_{l})=\left\{\begin{array}{ll} \tau <o_{0}>, & \;\gamma_{l}=1,\\ \left[2\tau \gamma_{l}-\tau \right]<o_{\varsigma \left(2\tau \gamma_{l}-\tau -\left[2\tau \gamma_{l}-\tau \right]\right)}>, & \;1<2\tau \gamma_{l}-\tau <\tau ,\\ 0<o_{\varsigma \left(2\tau \gamma_{l}-\tau \right)}>, & \;-1<2\tau \gamma_{l}-\tau \leq 1,\\ \left[2\tau \gamma_{l}-\tau \right]+1\;<o_{\varsigma \left(2\tau \gamma_{l}-\tau -\left[2\tau \gamma_{l}-\tau \right]-1\right)}>, & \;-\tau <2\tau \gamma_{l}-\tau <-1,\;\\ -\tau <o_{0}>, & \;\gamma_{l}=0.\; \end{array}\right. \end{array}$$

*As a result, the conversion functions F and F^−1^ between the DHHFLE*$h_{S_{O}}$*and the hesitant fuzzy linguistic element*$h_{\gamma }$*can be expressed as follows:*

(6) $$\begin{array}{ll} F:\Phi & \times \;\Psi \to \Theta ,\;\\ F\left(h_{S_{O}}\right) & =F\left(\left\{s_{\phi_{l}\langle o_{\varphi_{l}}\rangle }\left|s_{\phi_{l}\langle o_{\varphi_{l}}\rangle }\in {\bar{S}}_{O};l=1,\ldots ,L;\phi_{l}\in \left[-\tau ,\tau \right];\varphi_{l}\in \left[-\varsigma ,\varsigma \right]\right.\right\}\right)\\ & \;=\left\{\gamma_{l}\left|\gamma_{l}\right.=f\left(\phi_{l},\varphi_{l}\right);\;l=1,2,\ldots ,L\;\right\}\;=h_{\gamma }, \end{array}$$

(7) $$\begin{array}{ll} F^{-1}:\Theta & \to \Phi \times \Psi ,\;\\ \;F^{-1}\left(h_{\gamma }\right) & =F^{-1}\left(\left\{\gamma_{l}\left|\gamma_{l}\in \left[0,1\right];\;l=1,2,\ldots ,\right.L\right\}\right)\\ & \;=\left\{s_{\phi_{l}\langle o_{\varphi_{l}}\rangle }\left|\phi_{l}\right.\langle o_{\varphi l}\rangle =f^{-1}(\gamma_{l})\;\right\}\;=h_{S_{O}}. \end{array}$$

*where* Φ × Ψ *is the set of all possible DHHFLEs, and* Θ *be the set of all numerical scales.*

**Definition** **4** **([31]).**  *For any three DHHFLEs* $h_{S_{O}}$ *,* $h_{S_{O_{1}}}$ *and* $h_{S_{O_{2}}}$ *, and let* λ *be a real number, the operational laws of DHHFLEs are defined as follows:*

*(1)*  $h_{S_{O_{1}}}⊕h_{S_{O_{2}}}=F^{-1}\left(\underset{\eta_{1}\in F\left(h_{S_{O_{1}}}\right),\eta_{2}\in F\left(h_{S_{O_{2}}}\right)}{\cup }\left\{\eta_{1}+\eta_{2}-\eta_{1}\eta_{2}\right\}\right);$
*(2)*  $h_{S_{O_{1}}}⊗h_{S_{O_{2}}}=F^{-1}\left(\underset{\eta_{1}\in F\left(h_{S_{O_{1}}}\right),\eta_{2}\in F\left(h_{S_{O_{2}}}\right)}{\cup }\left\{\eta_{1}\eta_{2}\right\}\right);$
*(3)*  $\lambda h_{S_{O}}=F^{-1}\left(\underset{\eta \in F\left(h_{S_{O}}\right)}{\cup }\left\{1-{\left(1-\eta \right)}^{\lambda }\right\}\right);$
*(4)*  ${\left(h_{S_{O}}\right)}^{\lambda }=F^{-1}\left(\underset{\eta \in F\left(h_{S_{O}}\right)}{\cup }\left\{\eta^{\lambda }\right\}\right).$

**Definition** **5** **([31]).**  *Let* $S_{O}$ *be a DHLTS and* $h_{S_{O}}\left(x_{i}\right)$ *be a DHHFLE. The expected function of* $h_{S_{O}}$ *is represented as*

(8) $$E\left(h_{S_{O}}\right)=\frac{1}{L}\sum_{l=1}^{L}F\left(s_{\phi_{l}\langle o_{\varphi l}\rangle }\right),$$

*and the variance of* $h_{S_{O}}$ *is defined as*

(9) $$\upsilon \left(h_{S_{O}}\right)=\sqrt{\frac{1}{L}{\sum_{l=1}^{L}\left(F\left(s_{\phi_{l}\langle o_{\varphi_{l}}\rangle }\right)-E\right)}^{2}}.$$

**Definition** **6** **([31]).**  *Let* $h_{S_{O_{1}}}$ *and* $h_{S_{O_{2}}}$ *be any two DHHFLEs, then*

(1) If $E\left(h_{S_{O_{1}}}\right)>E\left(h_{S_{O_{2}}}\right)$, then $h_{S_{O_{1}}}$ is superior to $h_{S_{O_{2}}}$, denoted by $h_{S_{O_{1}}}>h_{S_{O_{2}}}$;
(2) If $E\left(h_{S_{O_{1}}}\right)=E\left(h_{S_{O_{2}}}\right)$, then
(3) If $V\left(h_{S_{O_{1}}}\right)<V\left(h_{S_{O_{2}}}\right)$, then $h_{S_{O_{1}}}$ is superior to $h_{S_{O_{2}}}$, denoted by $h_{S_{O_{1}}}>h_{S_{O_{2}}}$;
(4) If $V\left(h_{S_{O_{1}}}\right)=V\left(h_{S_{O_{2}}}\right)$, then $h_{S_{O_{1}}}$ is equal to $h_{S_{O_{2}}}$, denoted by $h_{S_{O_{1}}}=h_{S_{O_{2}}}$.

**Definition** **7** **([31]).**  *Let*$S_{O}$*be a DHLTS,*$h_{S_{O_{1}}}$*and*$h_{S_{O_{2}}}$*be any two DHHFLEs. Then, the Euclidean distance between*$h_{S_{O_{1}}}$*and*$h_{S_{O_{2}}}$*by*

(10) $$d\left(h_{S_{O_{1}}},h_{S_{O_{2}}}\right)=(\frac{1}{L}\underset{\eta^{2}{}_{\sigma \left(l\right)}\in \;F\left(h_{S_{O_{2}}}\right)}{\underset{\eta^{1}{}_{\sigma \left(l\right)}\in \;F\left(h_{S_{O_{1}}}\right)}{\sum_{l=1}^{L}}}{\left(\left|\eta^{1}{}_{\sigma \left(l\right)}-\eta^{2}{}_{\sigma \left(l\right)}\right|\right)}^{2}{)}^{1/2},$$

*where*$\eta^{1}{}_{\sigma \left(l\right)}$*and*$\eta^{2}{}_{\sigma \left(l\right)}$*are the lth largest elements of*$F\left(h_{S_{O_{1}}}\right)$*and*$F\left(h_{S_{O_{2}}}\right)$*, respectively. Note that If the number of elements in*$F\left(h_{S_{O_{1}}}\right)$*is not equal to the number of elements in*$F\left(h_{S_{O_{2}}}\right)$, *we can extend the shorter one with the average value of its upper and lower bounds [37,38].*

**Definition** **8.**  *Let*$h_{S_{O_{i}}}\left(i=1,2,\ldots n\right)$*be a set of DHHFLEs, and*$w_{i}\in \left[0,1\right]$*satisfying*$\sum_{i=1}^{n}w_{i}=1$. *Then the double hierarchy hesitant fuzzy linguistic weighted geometric (DHHFLWG) operator is defined as:*

(11) $$\begin{array}{ll} DHHFLWG\left(h_{S_{O_{1}}},h_{S_{O_{2}}},\ldots h_{S_{O_{n}}}\right) & =\overset{n}{\underset{i=1}{⊕}}{\left(h_{S_{O_{i}}}\right)}^{w_{i}}\\ & \;=F^{-1}\left\{\underset{\eta_{i}\in F\left(h_{S_{O_{i}}}\right)}{\cup }\left[\left(1-{\prod_{i=1}^{n}\left(1-\eta_{i}^{1}\right)}^{w_{i}}\right),\left(1-{\prod_{i=1}^{n}\left(1-\eta_{i}^{2}\right)}^{w_{i}}\right),\ldots ,\left(1-{\prod_{i=1}^{n}\left(1-\eta_{i}^{l}\right)}^{w_{i}}\right)\right]\right\}, \end{array}$$

*where l is themaximum number ofelements in DHHFLEs*$h_{S_{O_{i}}}\left(i=1,2,\ldots ,n\right)$.

## 3. The Proposed DHHFL–DEMATEL Approach

**Figure 1:**
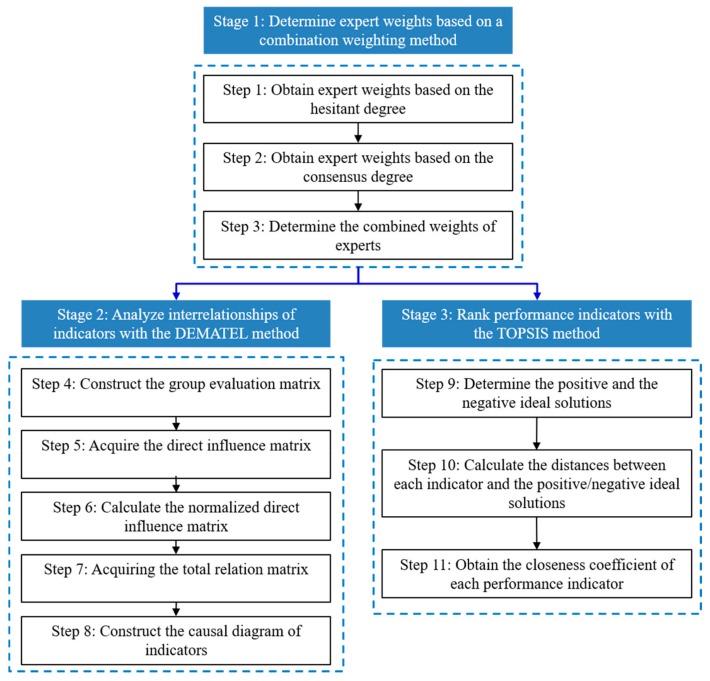
Flowchart of the proposed DHHFL–DEMATEL approach.

In this part, an integrated decision-making approach based on the DHHFLTSs and DEMATEL method is developed to analyze the inter-relationships of healthcare performance indicators and identify KPIs for hospital management. In this approach, the DHHFLTSs are utilized to represent the evaluations of domain experts on the inter-relationships between indicators. The DEMATEL method is utilized to identify causal relationships between indicators as well as to define the KPIs. Also, the importance of healthcare performance indicators is ranked using a modified TOPSIS method. Figure 1 shows the procedure of the proposed DHHFL–DEMATEL approach.

To determine KPIs in the healthcare performance management, suppose that $F=\left\{F_{1},F_{2},\ldots ,F_{n}\right\}$ is a set of performance indicators, $E=\left\{E_{1},E_{2},\ldots ,E_{m}\right\}$ is a set of experts. Let $H_{}^{k}={\left(h_{S_{O_{ij}}}^{k}\right)}_{n\times n}\left(k=1,2,\ldots ,m\right)$ be the hesitant linguistic evaluation matrix of the *k*th expert on the interdependence between indicators, where $h_{S_{O_{ij}}}^{k}$ is a DHHFLE provided by $E_{k}$ on the interrelation between indicators $F_{i}$ and $F_{j}$. In the following subsections, the proposed integrated approach consisting of three stages is described. 

### 3.1. Determine Expert Weights Based on a Combination Weighting Method

According to [39,40,41], the greater the hesitant degree of evaluation information, the lower the reliability could be. Thus, the expert with higher hesitant degree should be endowed with a lower weight. In addition, the consensus degree of assessment information is also considered in many studies for obtaining experts’ weights [15,42,43]. This method can not only reduce the influence of biased opinions on the final decision-making results, but also obtain the collective evaluations which are acceptable to most experts [43]. In this stage, a combination weight method based on both hesitant degree and consensus degree is proposed to determine the relative weights of the *m* experts. Its specific steps are listed as follows.

**Step 1:** Obtain expert weights based on the hesitant degree

The weight of expert $E_{k}$ based on the hesitant degree can be computed by

(12) $$w_{k}^{}=\frac{1-HD\left(H_{}^{k}\right)}{\sum_{k=1}^{m}\left(1-HD\left(H_{}^{k}\right)\right)},$$

(13) $$HD\left(H_{}^{k}\right)=\frac{1}{n\times n}\sum_{i=1}^{n}\sum_{j=1}^{n}\left(1-\frac{1}{\#h_{S_{O_{ij}}}}\right),$$

*where* $HD\left(H_{}^{k}\right)$ *is the hesitant degree of* $E_{k}$ *and* $\#h_{S_{O_{ij}}}$ *represents the number of DHLTSs in* $h_{S_{O_{ij}}}^{k}$.

**Step 2:** Obtain expert weights based on the consensus degree

The weight of expert $E_{k}$ based on the consensus degree is calculated by

(14) $$\omega_{k}=\frac{C\left(H_{}^{k},\bar{H}\right)}{\sum_{k=1}^{m}C\left(H_{}^{k},\bar{H}\right)},$$

(15) $$C\left(H_{}^{k},\bar{H}\right)=1-d\left(H_{}^{k},\bar{H}\right)=1-\frac{1}{n\times n}{\sum_{i=1}^{n}\sum_{j=1}^{n}\left(\frac{1}{L}\sum_{\begin{array}{c} l=1\\ \eta_{\sigma \left(l\right)}^{k}\in \;F\left(H_{}^{k}\right),\\ {\bar{\eta }}_{\sigma \left(l\right)}^{}\in \;F\left(\bar{H}\right) \end{array}}^{L}{\left(\left|\eta_{\sigma \left(l\right)}^{k}-{\bar{\eta }}_{\sigma \left(l\right)}^{}\right|\right)}^{2}\right)}^{1/2}.$$

where $C\left(H_{}^{k},\bar{H}\right)$ is the consensus degree between $H_{}^{k}$ and $\bar{H}$, and $\bar{H}$ is the average evaluation matrix of the m evaluation matrices $H_{}^{k}\left(k=1,2,\ldots ,m\right)$. 

**Step 3:** Determine the combined weights of experts

In this step, we combine the above two types of weights to obtain the combined weights of experts. The combined expert weight $\lambda_{k}$ is calculated by

(16) $$\lambda_{k}=\alpha w_{k}+\left(1-\alpha \right)\omega_{k},$$

where α is a parameter indicating the relative importance between the experts’ hesitant and consensus weights, satisfying 0≤α≤1. 

### 3.2. Analyze Interrelationships of Indicators with the DEMATEL Method

In this stage, we use the DEMATEL method to analyze the interrelationships of healthcare performance indicators. The application steps are explained below. 

**Step 4:** Construct the group hesitant linguistic evaluation matrix

By applying the DHHFLWG operator, the group hesitant linguistic evaluation matrix $H={\left(h_{S_{O_{ij}}}^{}\right)}_{n\times n}$ is obtained, where

(17) $$\begin{array}{ll} h_{S_{O_{ij}}}^{} & =DHHFLWG\left(h_{S_{O_{ij}}}^{1},h_{S_{O_{ij}}}^{2},h_{S_{O_{ij}}}^{3},\ldots h_{S_{O_{ij}}}^{m}\right)=\overset{m}{\underset{k=1}{⊕}}{\left(h_{S_{O_{ij}}}^{k}\right)}^{\lambda_{k}}\\ & \;=F^{-1}\left\{\underset{\eta_{k}\in F\left(h_{{}_{S_{O}}}^{k}\right)}{\cup }\left[\left(1-\prod_{k=1}^{m}{\left(1-\eta_{k}^{1}\right)}^{\lambda_{k}}\right),\left(1-\prod_{k=1}^{m}{\left(1-\eta_{k}^{2}\right)}^{\lambda_{k}}\right),\ldots ,\left(1-\prod_{k=1}^{m}{\left(1-\eta_{k}^{l}\right)}^{\lambda_{k}}\right)\right]\right\}, \end{array}$$

where l is the maximum number of elements in DHHFLEs $h_{S_{O_{ij}}}^{k}\left(k=1,2,\ldots ,m\right)$.

**Step 5:** Acquire the direct influence matrix

The direct influence matrix $Z={\left(z_{ij}\right)}_{n\times n}$ is obtained by calculating the expected value of each element in the group hesitant linguistic evaluation matrix *H*. That is,

(18) $$z_{ij}=E\left(h_{S_{O_{ij}}}\right).$$

**Step 6:** Calculate the normalized direct influence matrix

After acquiring the direct influence matrix *Z*, the normalized direct influence matrix, denoted by $X={\left(x_{ij}\right)}_{n\times n}$, is computed by

(19) $$X=\frac{Z}{\max\left\{\underset{1\leq i\leq n}{\max}\sum_{j=1}^{n}z_{ij},\underset{1\leq j\leq n}{\max}\sum_{i=1}^{n}z_{ij}\right\}}.$$

**Step 7:** Compute the total influence matrix

The total influence matrix $T={\left(t_{ij}\right)}_{n\times n}$ is computed through

(20) $$T=\underset{\theta \to \infty }{\lim}\left(X+X_{}^{2}+X_{}^{3}+\cdots +X_{}^{\theta }\right)=X{\left(I-X\right)}^{-1},$$

in which *I* is represented as an n×n identity matrix.

**Step 8:** Construct the causal diagram of indicators

In this step, the sum of rows (*R*) and the sum of columns (*C*) from the total influence matrix *T* are obtained by using Equations (21) and (22).

(21) $$R={\left(r_{i}\right)}_{n\times 1}={\left(\sum_{j=1}^{n}t_{ij}\right)}_{n\times 1},$$

(22) $$C={\left(c_{j}\right)}_{1\times n}={\left(\sum_{i=1}^{n}t_{ij}\right)}_{1\times n},$$

where R represents the total influence that indicator $F_{i}$ exerts to the rest of the indicators while C represents the total influence that indicator $F_{i}$ receives from all the other indicators. 

By mapping the ordered pairs of $\left(R+C,\;R-C\right)$, we can draw a causal diagram of the *n* healthcare performance indicators. The value of R+C called “prominence” represents the strength of influences that are given and received of the indicators and the value of R−C named as “relation” shows the net effect contributed by the indicators. If R−C is positive, the indicator has a net influence on the other factors and can be grouped under the cause group; otherwise, the indicator is a net receiver and should be grouped under the effect group.

### 3.3. Rank Performance Indicators with the TOPSIS Method

In this stage, we use the TOPSIS method [44] to determine the ranking of the considered healthcare performance indicators. The principle is formulating the positive ideal solution and the negative ideal solution and then applying distance measure to find the solution that is closest to the ideal solution and farthest from the negative ideal solution [45,46]. Next, the steps of the TOPSIS method are explained. 

**Step 9:** Determine the positive and the negative ideal solutions

The best possible DHHFLTS and the worst possible DHHFLTS represent the most desirable indicator and the least desirable indicator, respectively, which can be defined by

(23) $$F^{+}=\left\{\underset{i}{\max}\left(h_{S_{O_{ij}}}\right)\right\}=\left\{F_{1}{}^{+},F_{2}{}^{+},\ldots ,F_{n}{}^{+}\right\},$$

(24) $$F^{-}=\left\{\underset{i}{\min}\left(h_{S_{O_{ij}}}\right)\right\}=\left\{F_{1}{}^{-},F_{2}{}^{-},\ldots ,F_{n}{}^{-}\right\}.$$

If $F_{i}\left(i=1,2,\ldots n\right)$ has the maximum expected value of $h_{S_{O_{ij}}}$, it means $F_{i}$ is the best possible DHHFLTS, denoting by $F_{i}^{+}$. By contrast, if $F_{i}\left(i=1,2,\ldots n\right)$ has the minimum expected value of $h_{S_{O_{ij}}}$, it means $F_{i}$ is the worst possible DHHFLTS, denoting by $F_{i}^{-}$.

**Step 10:** Calculate the distances between each indicator and the positive/negative ideal solutions

The distances from $F_{i}$ to the positive ideal solution and the negative ideal solution are calculated by Equations (25) and (26).

(25) $$d_{i}{}^{+}=d\left(F_{i},F^{+}\right)=\sqrt{\sum_{i=1}^{n-1}\sum_{j=i+1}^{n}\frac{1}{L}\sum_{\begin{array}{c} l=1\\ \eta_{\sigma \left(l\right)}^{1}\in \;F_{i}\\ \eta_{\sigma \left(l\right)}^{2}\in \;F^{+} \end{array}}^{L}{\left(\left|\eta_{\sigma \left(l\right)}^{1}-\eta_{\sigma \left(l\right)}^{2}\right|\right)}^{2}},$$

(26) $$d_{i}{}^{-}=d\left(F_{i},F^{-}\right)=\sqrt{\sum_{i=1}^{n-1}\sum_{j=i+1}^{n}\frac{1}{L}\sum_{\begin{array}{c} l=1\\ \eta_{\sigma \left(l\right)}^{1}\in F_{i}\;\\ \eta_{\sigma \left(l\right)}^{2}\in F^{-}\; \end{array}}^{L}{\left(\left|\eta_{\sigma (l)}^{1}-\eta_{\sigma (l)}^{2}\right|\right)}^{2}}.$$

**Step 11:** Obtain the closeness coefficient of each performance indicator

For each of the *n* healthcare performance indicators, its closeness coefficient to the positive ideal solution is calculated by

(27) $$C_{i}^{*}=\frac{D_{i}{}^{-}}{D_{i}{}^{+}+D_{i}{}^{-}},\;i=1,2,\ldots ,n.$$

The larger the closeness coefficient value $C_{i}^{*}$, the greater the influence of the indicator $F_{i}$. Thus, all the indicators can be sorted according to the descending order of their closeness coefficient values $C_{i}^{*}\left(i=1,2,\ldots ,n\right)$. In other words, the indicators with the higher $C_{i}^{*}$ values are considered to be non-negligible and particularly important. 

## 4. Illustrative Example

In this section, a rehabilitation hospital located in Shanghai, China is used as an example to show the flexibility and effectiveness of our proposed DHHFL–DEMATEL approach for identifying healthcare KPIs. 

### 4.1. Application

The considered hospital is a tertiary care university teaching hospital, and provides the services of medical treatment, teaching, scientific research, first aid, rehabilitation, prevention, and healthcare. There are 39 clinical and medical technique departments, and 16 of them are key disciplines. Up to now, the hospital has nearly 1800 employees with medical professionals accounting for 80%. Meanwhile, the hospital has more than 900,000 emergency patients per year and 12,000 inpatients. In accordance with the requirements for tertiary hospital management standards and real healthcare needs, hospital administrators have decided to determine healthcare KPIs to improve the quality of hospital services. Therefore, the proposed integrated approach is applied to identify KPIs, then through the improvement of those KPIs to continuously enhance the healthcare quality of this hospital.

Via a systematic literature review on healthcare performance assessment, the performance indicators frequently used were identified in [1]. In this case study, 14 performance indicators shown in Table 1 were considered and analyzed to identity KPIs. To measure inter-relationships among the indicators, a group composed of five experts $E=\left\{E_{1},E_{2},\ldots ,E_{5}\right\}$ was established. The five experts adopt the linguistic term sets S and O for the evaluation of the direct relation of these influential indicators. Specifically, S is the linguistic term set of seven labels while O is the linguistic term set of five labels, and they are defined as follows:

$$\begin{array}{l} S=\left\{s_{-3}=no\;influence,s_{-2}=very\;low\;influence\;,s_{-1}=Low,\right.s_{0}=medium,\;s_{1}=high\;,s_{2}=very\;high,\left.s_{3}=extremely\;high\;\right\}\\ O=\left\{o_{-2}=far\;from,o_{-1}=a\;little,o_{0}=just\;right,o_{1}=much,o_{2}=entirely\right\}. \end{array}$$

As a result, the linguistic evaluations of the five experts are obtained. For example, the hesitant linguistic evaluation matrix $H_{}^{1}={\left(h_{S_{O_{ij}}}^{1}\right)}_{14\times 14}$ of the first expert is listed in Table 2. 

In what follows, the proposed DHHFL–DEMATEL approach is applied to the case study and the results are summarized.

**Step 1:** Based on Equations (12) and (13), the expert weight vector based on hesitant degree is computed as $w={\left(0.199,0.206,0.203,0.194,0.199\right)}^{T}$.

**Step 2:** By Equations (14) and (15), the expert weight vector based on consensus degree is calculated as $\omega ={\left(0.200,0.196,0.201,0.203,0.200\right)}^{T}$.

**Step 3:** Using Equation (16) and letting α be 0.5, the combined expert weights are calculated as: $\lambda_{1}=0.200,$ $\lambda_{2}=0.201,$ $\lambda_{3}=0.202,$ $\lambda_{4}=0.198,$ and $\lambda_{5}=0.199.$

**Step 4:** Applying Equation (17), we obtain the group hesitant linguistic evaluation matrix $H={\left(h_{S_{O_{ij}}}^{}\right)}_{14\times 14}$ as shown in Table 3.

**Step 5:** The direct influence matrix $Z={\left(z_{ij}\right)}_{14\times 14}$ is established through Equation (18), as presented in Table 4.

**Step 6:** The normalized direct influence matrix $X={\left(x_{ij}\right)}_{14\times 14}$ is constructed by Equation (19), as shown in Table 5.

**Step 7:** The total influence matrix $T={\left(t_{ij}\right)}_{14\times 14}$ is acquired by Equation (20), and presented in Table 6.

**Figure 2:**
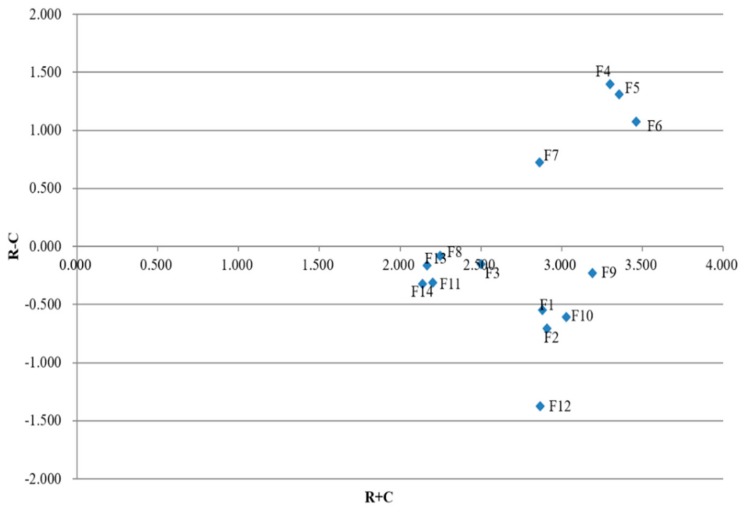
Causal diagram of the example.

**Step 8:** By using Equations (21) and (22), the sum of rows *R* and the sum of columns *C* of the matrix *T* are calculated as listed in Table 7. Then, based on the values of R+C and R−C, a causal diagram of the 14 healthcare performance indicators is draw as shown in Figure 2.

**Step 9:** The positive ideal solution and the negative ideal solution are identified as

$$A^{+}=\left\{\begin{array}{l} \left(S_{3\langle O_{0}\rangle }\right),\left(S_{3\langle O_{0}\rangle }\right),\left(S_{3\langle O_{0}\rangle }\right),\left(S_{2\langle O_{0.492}\rangle },S_{3\langle O_{0}\rangle }\right),\left(S_{2\langle O_{0.858}\rangle },S_{3\langle O_{0}\rangle }\right),\left(S_{1\langle O_{1.789}\rangle },S_{3\langle O_{0}\rangle }\right),\left(S_{3\langle O_{0}\rangle }\right),\\ \left(S_{3\langle O_{0}\rangle }\right),\left(S_{3\langle O_{0}\rangle }\right),\left(S_{3\langle O_{0}\rangle }\right),\left(S_{1\langle O_{0.770}\rangle },S_{1\langle O_{1.591}\rangle }\right),\left(S_{3\langle O_{0}\rangle }\right),\left(S_{2\langle O_{0.858}\rangle }\right),\left(S_{3\langle O_{0}\rangle }\right) \end{array}\right\}$$

$$A^{-}=\left\{\begin{array}{l} \left(S_{-1\langle O_{-1.632}\rangle },S_{0\langle O_{-1.407}\rangle }\right),\left(S_{-2\langle O_{-1.387}\rangle }\right),\left(S_{-2\langle O_{-1.189}\rangle },S_{-2\langle O_{-0.749}\rangle }\right),\left(S_{-2\langle O_{-0.734}\rangle },S_{-2\langle O_{-1.372}\rangle }\right),\left(S_{-2\langle O_{-1.170}\rangle }\right),\left(S_{-2\langle O_{-0.754}\rangle },S_{-2\langle O_{-0.505}\rangle }\right),\left(S_{-2\langle O_{-0.789}\rangle }\right),\\ \left(S_{-2\langle O_{-0.997}\rangle },S_{-2\langle O_{-1.192}\rangle }\right),\left(S_{-1\langle O_{-1.254}\rangle }\right),\left(S_{-2\langle O_{-0.082}\rangle }\right),\left(S_{-2\langle O_{-0.368}\rangle },S_{-1\langle O_{-1.958}\rangle }\right),\left(S_{0\langle O_{0.404}\rangle },S_{0\langle O_{0.511}\rangle }\right),\left(S_{-2\langle O_{-1.191}\rangle },S_{-2\langle O_{-0.982}\rangle }\right),\left(S_{-2\langle O_{-0.997}\rangle }\right) \end{array}\right\}$$

**Step 10:** Applying Equations (25) and (26), the distances between each indicator and the positive/negative solutions, $D_{i}{}^{+}$ and $D_{i}{}^{-}\left(i=1,2,\ldots ,14\right)$, are computed as listed in Table 8.

**Step 11:** Using Equation (27), the closeness coefficients of the 14 indicators $C_{i}^{*}\left(i=1,2,\ldots ,14\right)$ are computed and displayed in Table 8. As a consequence, the ranking result of the indicators is obtained in line with the descend order of their closeness coefficient values. 

### 4.2. Discussions

Based on the values of R−C, we can divide the 14 indicators into a cause group and an effect group. As shown in Table 7, the cause group is composed of $F_{4}$, $F_{5}$, $F_{6}$, and $F_{7}$, and the effect group includes $F_{1}$, $F_{2}$, $F_{3}$, $F_{8}$, $F_{9}$, $F_{10}$, $F_{11}$, $F_{12}$, $F_{13}$, and $F_{14}$. To determine KPIs, we first conducted the initial screening of the 14 performance indicators based on the ranking in Table 8. Next, we only analyzed the top seven indicators, which include four cause indicators (*F*_4_, *F*_5_, *F*_6_, *F*_7_) and three effect indicators (*F*_3_, *F*_9_, *F*_10_). 

Since cause indicators have a net impact on the whole system, their performance can seriously affect the whole healthcare system. Among the considered four cause indicators,$F_{4}$ has the largest value of R−C, which means that this indicator exerts an important influence on the system and is able to actively influence the other indicators. Therefore, $F_{4}$ is a KPI in the hospital management. With respect to $F_{6}$, its net effect value R−C ranks second among the cause indicators, and it has the highest value of R+C. That is, $F_{6}$ has a great impact on other indicators and optimization of $F_{6}$ can greatly improve the whole healthcare system. Hence, $F_{6}$ is recognized as a KPI. Similarly, $F_{5}$ can be regarded as a KPI. Although $F_{7}$ ranks fourth among the indicators, its R and C are not sufficiently high. The low value of R+C indicates that $F_{7}$ cannot have a notable impact on the improvement of the healthcare system. Thus, $F_{7}$ cannot be classified as a KPI. 

Normally, effect indicators are influenced by other indicators. However, it is necessary to analyze the effect indicators which can lead to the improvement of hospital management. The indicator *F*_9_ ranks fifth among all the indicators, revealing that it is comparatively significant in the hospital management. Furthermore, both R and C values of $F_{9}$ are high although its R−C value is negative. So, $F_{9}$ is identified as a KPI. According to the causal diagram of Figure 2, the R−C value of $F_{3}$ is slightly less than 0, meaning that $F_{3}$ is less affected by other indicators. And its importance degree of R+C is 2.507, which is not high enough to label it as a KPI. For the indicator $F_{10}$, its R+C value is as high as 3.034, but its value of R−C is −0.609, which indicates its very low importance in the system. To further elucidate this scenario, as we can see in Table 7, the influential impact degree of $F_{10}$ is low, and the C value is much larger than R. Thus, $F_{10}$ has no significant effect on the other indicators and is not a KPI.

## 5. Conclusions

In this study, we presented a new hybrid decision making approach by combing DHHFLTSs with the DEMATEL method for identifying KPIs in healthcare management. In this approach, the DHHFLTSs are employed to express the hesitancy and uncertainty of experts’ evaluations regarding the inter-relations between indicators. A combination weighting method was proposed to calculate expert weights considering their hesitant degree and consensus degree. To identify KPIs, the DEMATEL method was applied to divide performance indicators into cause and effect groups, and a TOPSIS method used to determine the ranking of the indicators. Finally, a practical example of a rehabilitation hospital is provided to validate the developed DHHFL–DEMATEL approach. According to the results of this case study, “Incidents/Errors”, “Accidents/Adverse events”, “Nosocomial infection”, and “Length of stay” are identified as KPIs for the healthcare performance management.

Despite its advantages, the proposed approach has the following limitations, which may be addressed by future researches. First, input of the DHHFL–DEMATEL method is based on the opinions of a small number of experts. This may make it hard to reflect the real situation of a complicated healthcare system. In the future, a large expert sample is suggested to assess performance indicators and obtain more generalizable results. Second, the proposed approach is unable to cope with experts’ incomplete assessments of performance indicators. Considering the lack of data, future research can find advanced uncertainty theories to handle KPI evaluation problems under the incomplete information environment, which is closer to the practice. Third, the proposed approach is only a prototype and should be improved by future research. For example, the dynamic DEMATEL model can be introduced to refine the identified factors, and validation of the identified KPIs based on simulation technique is necessary.

## Figures and Tables

**Table 1:** Performance indicators considered in this case study.

Indicators	Description
Overall satisfaction (*F*_1_)	Satisfaction with healthcare service, including satisfaction with physicians, wait time etc.
Overall complaint (*F*_2_)	Number of patients expressing complaint
Patient medical expenses (*F*_3_)	Per capita medical expenses for patients include hospitalization expenses, outpatient expenses, etc.
Incidents/Errors (*F*_4_)	Incidents/errors occurred in healthcare treatment process, including medication errors, dignosis, etc.
Accidents/Adverse events (*F*_5_)	Accident/adverse events occurring in healthcare treatment process
Nosocomial infection (*F*_6_)	Nosocomial infection in the hospital
Percentage of readmissions (*F*_7_)	Ratio of readmissions within 40 days of discharge, related to the same medical problem
Mortality/Death (*F*_8_)	Mortality/Death in healthcare organization
Length of stay (*F*_9_)	Time that the patient passes in hospital from the entrance to the exit
Bed occupancy Ratio (*F*_10_)	Average percentage occupancy of hospital beds
Waiting time (*F*_11_)	Total of time that a patient waits for an initial rehabilitation service
Net profit margin (*F*_12_)	Total operating revenue-total operating expenses/total operating revenue
Staff satisfaction (*F*_13_)	Number of staffs expressed “satisfaction”
Employee turnover (*F*_14_)	The turnover rate of employees leaving the hospital

**Table 2:** Hesitant linguistic evaluation matrix of the first expert.

Indicators	*F*_1_	*F*_2_	*F*_3_	…	*F*_13_	*F*_14_
*F*_1_	-	(S_1<O0>,_ S_2<O1>_)	(S_0<O0>,_ S_1<O-1>_)	…	(S_0<O0>_)	(S_-2<O-1>_)
*F*_2_	(S_3<O0>_)	-	(S_-2<O-1>_)	…	(S_-1<O2>,_ S_1<O0>_)	(S_0<O1>,_ S_1<O1>_)
*F*_3_	(S_2<O-1>,_ S_2<O0>_)	(S_1<O1>,_ S_2<O0>_)	-	…	(S_-2<O-1>_)	(S_-2<O-1>_)
*F*_4_	(S_2<O1>_)	(S_3<O0>_)	(S_1<O1>_)	…	(S_1<O1>_)	(S_1<O1>,_ S_2<O1>_)
*F*_5_	(S_2<O2>_)	(S_3<O-1>_)	(S_1<O1>,_ S_2<O1>_)	…	(S_0<O1>_)	(S_1<O1>_)
*F*_6_	(S_3<O-1>_)	(S_2<O1>_)	(S_2<O1>_)	…	(S_0<O-1>_)	(S_2<O0>_)
*F*_7_	(S_-1<O1>,_ S_0<O-1>_)	(S_1<O0>_)	(S_3<O-1>_)	…	(S_-2<O-1>_)	(S_-2<O-1>,_ S_-2<O0>_)
*F*_8_	(S_2<O0>_)	(S_1<O1>,_ S_2<O1>_)	(S_-1<O1>_)	…	(S_-1<O-1>_)	(S-_2<O0>_)
*F*_9_	(S_0<O0>,_ S_1<O-1>_)	(S_0<O0>,_ S_1<O0>_)	(S_2<O1>,_ S_3<O0>_)	…	(S_-2<O-1>_)	(S-_3<O1>_)
*F*_10_	(S_-1<O1>,_ S_1<O-2>_)	(S_1<O-1>_)	(S_1<O0>_)	…	(S_-3<O0>,_ S_-3<O1>_)	(S_-3<O0>,_ S_-2<O-1>_)
*F*_11_	(S_1<O1>,_ S_2<O-1>_)	(S_2<O1>_)	(S_-2<O-1>,_ S_-1<O0>_)	…	(S_0<O-1>,_ S_1<O1>_)	(S-_2<O0>_)
*F*_12_	(S_0<O-1>_)	(S_-2<O-1>_)	(S_0<O-1>_)	…	(S_1<O0>,_ S_2<O0>_)	(S_1<O-1>,_ S_2<O1>_)
*F*_13_	(S_0<O0>,_ S_0<O1>_)	(S_0<O-1>_)	(S_-2<O-1>,_ S_-1<O-1>_)	…	-	(S_2<O0>,_ S_2<O1>_)
*F*_14_	(S_0<O0>,_ S_1<O-1>_)	(S_0<O1>_)	(S_-3<O1>_)	…	(S_2<O1>_)	-

**Table 3:** The group hesitant linguistic evaluation matrix.

Indicators	*F*_1_	*F*_2_	*F*_3_	…	*F*_13_	*F*_14_
*F*_1_	-	(S_2<O0.483>_, S_2<O1>_)	(S_-1<O-0.056>_, S_0<O-1.284>_)	…	(S_0<O-1.211>,_ S_0<O-0.924>_)	(S_-1<O-0.247>,_ S_0<O-1.997>_)
*F*_2_	(S_3<O0>_)	-	(S_-2<O-0.157>_, S_-1<O-0.363>_)	…	(S_0<O-0.479>,_ S_0<O0.238>_)	(S_0<O0.454>,_ S_1<O0.243>_)
*F*_3_	(S_1<O0.099>_, S_1<O1.704>_)	(S_0<O1.380>_, S_1<O0.153>_)	-	…	(S_-2<O-0.775>,_ S_-2<O-0.170>_)	(S_-2<O-1>_)
*F*_4_	(S_3<O0>_)	(S_3<O0>_)	(S_1<O0.871>_)	…	(S_0<O1.907>,_ S_1<O0.276>_)	(S_0<O1.520>,_ S_1<O1.767>_)
*F*_5_	(S_3<O0>_)	(S_3<O0>_)	(S_3<O0>_)	…	(S_0<O-0.243>,_ S_0<O0.161>_)	(S_1<O1.721>,_ S_0<O1.961>_)
*F*_6_	(S_2<O0.680>_, S_3<O0>_)	(S_3<O0>_)	(S_2<O0.679>_, S_2<O1>_)	…	(S_0<O0.276>,_ S_0<O0.528>_)	(S_1<O0.342>,_ S_1<O0.570>_)
*F*_7_	(S_0<O0.071>_, S_0<O1.829>_)	(S_0<O1.801>_, S_1<O1.556>_)	(S_2<O0.111>_, S_2<O0.480>_)	…	(S_-2<O-1.194>,_ S_-2<O-0.984>_)	(S_-2<O-0.170>,_ S_-1<O-1.979>_)
*F*_8_	(S_0<O1.978>_, S_1<O0.136>_)	(S_0<O0.645>_, S_0<O1.699>_)	(S_-1<O0.997>_, S_-1<O-0.236>_)	…	(S_-1<O-1.638>_)	(S_-1<O-1.564>,_ S_-1<O-0.884>_)
*F*_9_	(S_0<O1.042>_, S_0<O1.867>_)	(S_0<O1.819>_, S_0<O1.594>_)	(S_2<O0.678>,_ S_3<O0>_)	…	(S_-2<O-0.052>,_ S_-1<O-0.485>_)	(S_-2<O-0.507>,_ S_-2<O0.092>_)
*F*_10_	(S_0<O0.066>_, S_0<O0.861>_)	(S_0<O-0.793>_)	(S_0<O-0.037>,_ S_0<O0.580>_)	…	(S_-2<O-1.132>,_ S_-2<O-0.544>_)	(S_-2<O-1.170>,_ S_-2<O-0.587>_)
*F*_11_	(S_1<O0.827>_, S_1<O1.454>_)	(S_1<O1.829>_, S_2<O0.113>_)	(S_-1<O-1.857>,_ S_0<O-1.912>_)	…	(S_-1<O-0.820>,_ S_0<O-0.110>_)	(S_-1<O-0.586>,_ S_-1<O-0.399>_)
*F*_12_	(S_-1<O-1.637>_, S_0<O1.411>_)	(S_-2<O-1.387>_)	(S_-1<O-1.862>,_ S_-1<O-1.675_)	…	(S_1<O1.370>,_ S_3<O0>_)	(S_2<O0.621>,_ S_2<O1>_)
*F*_13_	(S_0<O-1.686>_, S_0<O-0.521>_)	(S_-1<O-0.435>_, S_0<O-0.749>_)	(S_-2<O-1.191>,_ S_-2<O-0.752>_)	…	-	(S_3<O1>_)
*F*_14_	(S_0<O-1.488>,_ S_0<O-0.634->_)	(S_-1<O-0.154>_)	(S_-2<O-0.192>,_ S_-1<O-1.493>_)	…	(S_2<O0.850>_)	-

**Table 4:** The direct influencing matrix *Z*.

Indicators	*F*_1_	*F*_2_	*F*_3_	*F*_4_	*F*_5_	*F*_6_	*F*_7_	*F*_8_	*F*_9_	*F*_10_	*F*_11_	*F*_12_	*F*_13_	*F*_14_
*F*_1_	0.000	0.895	0.361	0.174	0.196	0.221	0.381	0.141	0.649	0.656	0.347	0.828	0.411	0.323
*F*_2_	1.000	0.000	0.228	0.268	0.163	0.189	0.391	0.083	0.553	0.752	0.406	0.860	0.490	0.612
*F*_3_	0.742	0.647	0.000	0.222	0.257	0.212	0.430	0.621	0.785	0.813	0.153	0.742	0.127	0.083
*F*_4_	1.000	1.000	0.739	0.000	0.952	0.908	1.000	1.000	1.000	0.921	0.579	1.000	0.674	0.720
*F*_5_	1.000	1.000	1.000	0.937	0.000	0.881	1.000	1.000	1.000	0.871	0.654	0.904	0.497	0.653
*F*_6_	0.945	1.000	0.903	0.835	0.928	0.000	0.958	0.945	0.937	0.813	0.640	0.937	0.533	0.705
*F*_7_	0.579	0.723	0.858	0.575	0.632	0.817	0.000	0.823	0.863	1.000	0.690	0.792	0.076	0.160
*F*_8_	0.671	0.598	0.282	0.311	0.418	0.279	0.206	0.000	0.490	0.574	0.378	0.657	0.197	0.231
*F*_9_	0.621	0.726	0.945	0.234	0.378	0.822	0.211	0.470	0.000	0.952	0.627	1.000	0.228	0.142
*F*_10_	0.539	0.434	0.523	0.123	0.197	0.795	0.211	0.420	0.869	0.000	0.764	0.892	0.097	0.093
*F*_11_	0.762	0.831	0.260	0.079	0.083	0.127	0.175	0.190	0.511	0.634	0.000	0.623	0.378	0.292
*F*_12_	0.290	0.051	0.186	0.110	0.069	0.114	0.101	0.075	0.316	0.551	0.273	0.000	0.890	0.901
*F*_13_	0.408	0.367	0.086	0.556	0.439	0.348	0.247	0.088	0.245	0.160	0.218	0.592	0.000	1.000
*F*_14_	0.412	0.321	0.180	0.410	0.499	0.163	0.110	0.118	0.229	0.182	0.326	0.538	0.904	0.000

**Table 5:** The normalized direct influencing matrix *X*.

Indicators	*F*_1_	*F*_2_	*F*_3_	*F*_4_	*F*_5_	*F*_6_	*F*_7_	*F*_8_	*F*_9_	*F*_10_	*F*_11_	*F*_12_	*F*_13_	*F*_14_
*F*_1_	0.000	0.078	0.031	0.015	0.017	0.019	0.033	0.012	0.056	0.057	0.030	0.072	0.036	0.028
*F*_2_	0.087	0.000	0.020	0.023	0.014	0.016	0.034	0.007	0.048	0.065	0.035	0.075	0.043	0.053
*F*_3_	0.065	0.056	0.000	0.019	0.022	0.018	0.037	0.054	0.068	0.071	0.013	0.065	0.011	0.007
*F*_4_	0.087	0.087	0.064	0.000	0.083	0.079	0.087	0.087	0.087	0.080	0.050	0.087	0.059	0.063
*F*_5_	0.087	0.087	0.087	0.082	0.000	0.077	0.087	0.087	0.087	0.076	0.057	0.079	0.043	0.057
*F*_6_	0.082	0.087	0.079	0.073	0.081	0.000	0.083	0.082	0.082	0.071	0.056	0.082	0.046	0.061
*F*_7_	0.050	0.063	0.075	0.050	0.055	0.071	0.000	0.072	0.075	0.087	0.060	0.069	0.007	0.014
*F*_8_	0.058	0.052	0.025	0.027	0.036	0.024	0.018	0.000	0.043	0.050	0.033	0.057	0.017	0.020
*F*_9_	0.054	0.063	0.082	0.020	0.033	0.072	0.018	0.041	0.000	0.083	0.055	0.087	0.020	0.012
*F*_10_	0.047	0.038	0.045	0.011	0.017	0.069	0.018	0.037	0.076	0.000	0.066	0.078	0.008	0.008
*F*_11_	0.066	0.072	0.023	0.007	0.007	0.011	0.015	0.017	0.044	0.055	0.000	0.054	0.033	0.025
*F*_12_	0.025	0.004	0.016	0.010	0.006	0.010	0.009	0.007	0.028	0.048	0.024	0.000	0.077	0.078
*F*_13_	0.035	0.032	0.007	0.048	0.038	0.030	0.022	0.008	0.021	0.014	0.019	0.052	0.000	0.087
*F*_14_	0.036	0.028	0.016	0.036	0.043	0.014	0.010	0.010	0.020	0.016	0.028	0.047	0.079	0.000

**Table 6:** The total influence matrix *T*.

Indicators	*F*_1_	*F*_2_	*F*_3_	*F*_4_	*F*_5_	*F*_6_	*F*_7_	*F*_8_	*F*_9_	*F*_10_	*F*_11_	*F*_12_	*F*_13_	*F*_14_
*F*_1_	0.059	0.128	0.073	0.044	0.048	0.058	0.065	0.047	0.109	0.115	0.071	0.139	0.074	0.070
*F*_2_	0.142	0.058	0.064	0.053	0.047	0.057	0.067	0.044	0.104	0.124	0.078	0.145	0.084	0.095
*F*_3_	0.125	0.113	0.048	0.050	0.055	0.062	0.072	0.091	0.126	0.134	0.060	0.139	0.051	0.050
*F*_4_	0.211	0.204	0.158	0.069	0.151	0.161	0.158	0.166	0.205	0.207	0.139	0.234	0.139	0.147
*F*_5_	0.211	0.204	0.178	0.143	0.073	0.158	0.158	0.166	0.205	0.204	0.144	0.226	0.123	0.140
*F*_6_	0.203	0.200	0.167	0.134	0.146	0.084	0.153	0.159	0.196	0.195	0.141	0.224	0.125	0.142
*F*_7_	0.149	0.155	0.146	0.098	0.107	0.134	0.059	0.134	0.168	0.185	0.128	0.183	0.069	0.079
*F*_8_	0.115	0.106	0.067	0.056	0.066	0.063	0.052	0.036	0.097	0.108	0.074	0.125	0.056	0.061
*F*_9_	0.133	0.136	0.137	0.060	0.075	0.119	0.066	0.090	0.078	0.161	0.108	0.178	0.072	0.068
*F*_10_	0.111	0.100	0.093	0.043	0.052	0.107	0.056	0.077	0.134	0.069	0.110	0.152	0.052	0.053
*F*_11_	0.113	0.115	0.057	0.031	0.033	0.043	0.042	0.044	0.089	0.104	0.035	0.113	0.066	0.061
*F*_12_	0.062	0.040	0.042	0.031	0.029	0.036	0.029	0.029	0.061	0.081	0.050	0.046	0.103	0.105
*F*_13_	0.089	0.082	0.047	0.077	0.070	0.065	0.054	0.043	0.072	0.068	0.057	0.113	0.041	0.124
*F*_14_	0.083	0.073	0.050	0.062	0.070	0.047	0.040	0.041	0.065	0.064	0.061	0.103	0.110	0.039

**Table 7:** Sum of influences given and received for each indicator.

Indicators	R	C	R + C	R − C	Cause/Effect
*F*_1_	1.103	1.809	2.912	−0.706	Effect
*F*_2_	1.167	1.717	2.884	−0.550	Effect
*F*_3_	1.177	1.329	2.507	−0.152	Effect
*F*_4_	2.351	0.953	3.305	1.398	Cause
*F*_5_	2.336	1.024	3.360	1.313	Cause
*F*_6_	2.270	1.195	3.464	1.075	Cause
*F*_7_	1.795	1.072	2.867	0.723	Cause
*F*_8_	1.086	1.168	2.253	−0.082	Effect
*F*_9_	1.484	1.713	3.197	−0.229	Effect
*F*_10_	1.213	1.822	3.034	−0.609	Effect
*F*_11_	0.948	1.259	2.207	−0.310	Effect
*F*_12_	0.746	2.126	2.872	−1.379	Effect
*F*_13_	1.003	1.168	2.170	−0.165	Effect
*F*_14_	0.908	1.234	2.142	−0.326	Effect

**Table 8:** Computation results by the TOPSIS method.

Indicators	$D_{i}{}^{+}$	$D_{i}{}^{-}$	$C_{i}^{*}$	Ranking
*F*_1_	2.083	1.277	0.380	9
*F*_2_	2.037	1.327	0.394	8
*F*_3_	2.030	1.349	0.399	6
*F*_4_	0.505	2.697	0.842	1
*F*_5_	0.579	2.690	0.823	2
*F*_6_	0.576	2.596	0.818	3
*F*_7_	1.423	2.056	0.591	4
*F*_8_	2.075	1.027	0.331	12
*F*_9_	1.790	1.792	0.500	5
*F*_10_	2.117	1.391	0.396	7
*F*_11_	2.326	1.166	0.334	11
*F*_12_	2.605	1.239	0.322	13
*F*_13_	2.339	1.194	0.338	10
*F*_14_	2.407	1.062	0.306	14
